# An Emergency Seamless Positioning Technique Based on ad hoc UWB Networking Using Robust EKF

**DOI:** 10.3390/s19143135

**Published:** 2019-07-16

**Authors:** Houzeng Han, Jian Wang, Fei Liu, Jixian Zhang, Deng Yang, Binghao Li

**Affiliations:** 1 School of Geomatics and Urban Spatial Informatics, Beijing University of Civil Engineering and Architecture (BUCEA), Beijing 102616, China; 2 School of Environment Science and Spatial Informatics, China University of Mining and Technology (CUMT), Xuzhou 221116, China; 3 National Quality Inspection and Testing Center for Surveying and Mapping Products, Beijing 100830, China; 4 School of Minerals and Energy Resources Engineering, The University of New South Wales (UNSW), Sydney 2053, Australia

**Keywords:** emergency positioning, UWB, robust EKF, indoor and outdoor seamless positioning

## Abstract

In this paper, a new emergency positioning technique is proposed based on ad hoc GNSS/UWB (Global Navigation Satellite System/Ultra-Wideband) network. The main innovations of the program are reflected in two aspects. First of all, a unified coordinate frame for indoor and outdoor environments is constructed dynamically with GNSS/UWB integration. In the outdoor environments, the high accuracy positioning can be achieved with GNSS/UWB equipment. The high-accuracy indoor coordinate is obtained by measuring the range observations between adjacent network nodes and outdoor GNSS/UWB nodes, and the range information of the UWB network is transmitted to the cloud server center. A network adjustment algorithm is proposed to improve the positioning accuracy of the UWB network. Secondly, a UWB indoor location algorithm based on robust EKF (Extended Kalman Filter) is proposed. By analyzing the transfer characteristics of gross error in EKF model, a new robust EKF model is established. The model is constructed based on the statistical characteristics of redundant observation components and prediction residual. The robust equivalent gain matrix is constructed, and the robust positioning solution of UWB is obtained with iteration. The global test is carried out first to further improve the real-time operation efficiency. Finally, a field indoor and outdoor seamless positioning experiment was carried out to verify the effectiveness of the proposed algorithm. The results show that the positioning accuracy of UWB emergency network nodes (anchors) can reach 0.35 m. Based on the network, the positioning accuracy of the tag can reach 0.38 m by applying the improved robust EKF positioning algorithm, which is improved by 20.83% and 73.43% compared with standard EKF and least square method, respectively.

## 1. Introduction

People live and work indoors more than 90% of their lives [1]. Every year, a large number of indoor people are killed and injured because of emergencies such as building fires [2], including many firefighters who lost their way or couldn’t find a safe exit in the fire. A high-precision, rapid deployment of indoor and outdoor seamless emergency positioning network is very important for indoor emergency rescue. In recent years, indoor and outdoor seamless positioning technology has become a research hotspot. The outdoor positioning method based on GNSS (Global Navigation Satellite System) can achieve sub-meter positioning, the indoor positioning method based on WLAN (Wireless Local Area Network) [3,4], WIFI(Wireless Fidelity) [5,6], Bluetooth [7,8], Smartphone [9,10], Vision [11,12], UWB (Ultra-Wideband) [13,14], and other sensors [15,16], also can acquire sub-meter location accuracy. However, a high-precision, rapid seamless positioning method is more difficult to achieve. Richter, P. et al. [17] propose a particle filter algorithm of fusing GNSS pseudo ranges and WLAN Signal Strengths, and achieves accurate and robust seamless localization with a median accuracy of five meters. And other seamless positioning methods based on GNSS and WLAN are also researched by [18,19,20]. However, the flexibility of those methods is limited by the pre-deployment of WLAN equipment. Guo, M. et al. [21] propose a positioning solution that is based on BeiDou satellite navigation system and radiofrequency technology in indoor and outdoor scenic spots. This method needs the pre-deployment radiofrequency spots. Cheng R.-S. et al. [22] proposes a system based on GPS(Global Positioning System), Bluetooth Low Energy (BLE) beacons, and Near Field Communication (NFC) technology. The experimental results confirm the ability of the proposed app to switch automatically from an outdoor mode to an indoor mode and to guide the user to requested target destination via the shortest possible route. Basiri, A. et al. [23] suggest seamless pedestrian positioning and navigation method with landmarks. Zou, H. et al. [24] propose BlueDetect as an accurate, fast response and energy-efficient scheme for IO detection and seamless LBS running on the mobile device based on the emerging low-power iBeacon technology, which provides 2.18 m on average in semi-outdoor areas with an enhancement of accuracy around 89.87%. Tanigawa, M. et al. [25] develop an experimental system using low-cost commercial off-the-shelf UWB positioning system to augment GPS/MEMS INS systems where UWB positioning infrastructure is determined with a tape measure. And a fusion algorithm employs a 15-state Kalman filter in a loosely-coupled architecture is adopted. Dynamic Position accuracy of 20 cm was obtained where UWB position measurements were available, and a tactical-grade IMU was used as reference. A seamless outdoor-to-indoor pedestrian navigation using GPS/UWB and Kalman filter is proposed by Chiu, D.S. of the University of Calgary. In their scheme, three UWB radios are set up in fixed and known positions inside the building, and the solution whilst indoors reported accuracies in the sub-meter level [26]. A cooperative positioning system based on GPS/UWB/MARG is proposed, which can achieve the seamless positioning between buildings in the hybrid scene. The average positioning accuracy of the system increased by 64% (from 8.9 m to 3.2 m), However, the UWB anchors need to be layout and the coordinates of them need to be measured in advance [27]. Tadic, S. at el. [28] put forward a localization of emergency technique using UWB/GNSS with cloud-based augmentation. In this research, a measurement-level GNSS/UWB integrated device is used as the reference station, and a low-cost GNSS/UWB device is used as the mobile station. The experimental data are transmitted back to the cloud platform through 3G/4G (the 3rd/4th Generation mobile communication technology) in real time to calculate the coordinates of the mobile station. Through the experimental test on the standard football field, the positioning result of the mobile station can reach 0.34 m. The seamless positioning technical methods base on GNSS, UWB and other sensors are put forward by [29,30,31]. Similar to other methods, the UWB anchors are needed to be prepared very well before the positioning activities. Sung, R. et al. [32] propose a sound-based indoor and outdoor environment detection method to realize seamless positioning handover for in-and-outdoor integrated positioning systems. This method is developed to detect indoor and outdoor environments for positioning systems, but it also needs a great deal of preparatory work and does not show the positioning accuracy. In addition, nonlinear algorithms such as EKF, UKF (Unscented Kalman Filter) and PF (Particle filter) are widely used in multi-sensor fusion localization algorithms. Although all three can solve the non-linear problem, UKF and PF have more advantages in the problem of high degree of non-linearity. In order to solve the problem of low degree of non-linearization, EKF has the characteristics of simple calculation model and less calculation, and also has a certain competitive advantage [33,34].

In summary, it can be seen that the existing technologies require two preconditions in the implementation process. First of all, it is necessary to set up the corresponding anchor equipment in advance, such as WIFI device, Bluetooth device, landmark and so on. Secondly, it is necessary to accurately determine the spatial position of each marker point. Therefore, the above methods have some problems, such as poor flexibility of random adjustment, large preparation workload, or low positioning accuracy.

In order to solve the above problems, this paper proposes an indoor and outdoor integrated seamless positioning technology based on GNSS/UWB network. Firstly, the GNSS/UWB base station equipment is used to quickly construct the indoor and outdoor integrated spatial reference, and the indoor and outdoor reference is unified to the GNSS system. Secondly, the UWB anchor equipment can be gradually placed from the outside to the inside without prior layout. The spatial coordinates of the anchor can achieve through the positioning network and cloud platform without prior measurement. Finally, based on the tag equipment and the cloud platform, personnel will be located when the ranges information transmit to the platform through 4G network in real-time, to achieve online monitoring of personnel. This method has the characteristics of fast layout, strong flexibility, and high positioning accuracy and so on.

The main contributions of this research are as follows. Firstly, a seamless location solution for rescue workers in emergency situations is put forward. The biggest advantage of this scheme is that it does not need to set up UWB anchors and measure their positions in advance, and the anchor coordinates are iterated to the interior through the outdoor positioning data, so this scheme is suitable for rapid deployment in emergency situations. Another is that an improved UWB localization algorithm for indoor location based on Robust EKF is proposed. The improved algorithm can adjust the size of gain matrix according to the predicted residuals, which can weaken or eliminate the influence of gross errors on the state vector.

This paper is organized as follows. In Section 2, the technical scheme and hardware equipment of the method are described in detail. In the Section 3, the networking positioning algorithm is introduced. In Section 4, the indoor and outdoor integrated positioning experiments are carried out, and the feasibility, flexibility and positioning accuracy of the method are analyzed. The last section summarizes the technical methods of this paper and gives the relevant conclusions.

## 2. The Seamless Emergency Positioning Scheme

### 2.1. Positioning System

**Figure 1:**
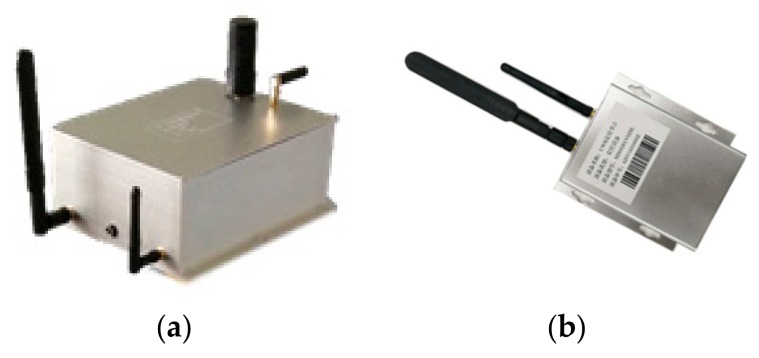
The indoor and outdoor seamless positioning devices. (**a**) Global Navigation Satellite System/Ultra-Wideband (GNSS/UWB) seamless positioning base device; (**b**) UWB anchor and tag two-in-one device.

**Figure 2:**
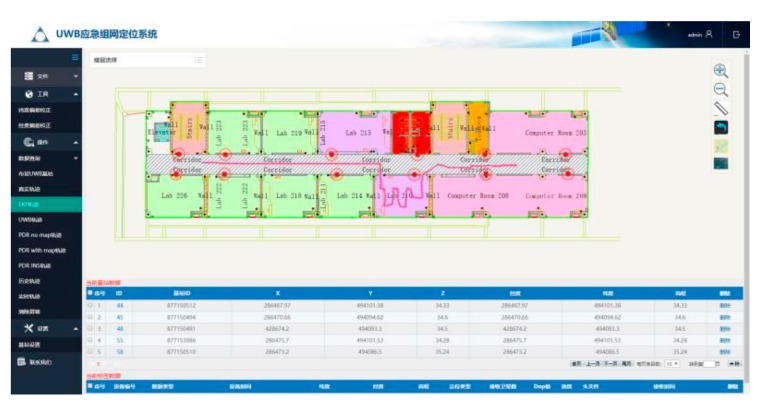
Personnel monitoring and dispatching system.

The positioning system includes positioning devices, personnel monitoring and dispatching system. As shown in Figure 1 and Figure 2.

The positioning equipment include GNSS/UWB base station, UWB anchor and tag. The base station, integrated with a GNSS receiver, a UWB positioning module, a 4G communication module, and a package structure, is used for acquiring absolute positioning datum at centimeter level. Usually, three or more make a group for 3-dimension coordinate calculation, two or more make a group for 2-dimension coordinate calculation, and two groups is necessary in an emergency positioning application. Its positioning information can be transmitted to the personnel monitoring and dispatching system with the 4G communication module in real time. The performance as shown in Table 1.

The UWB anchor and tag is a two-in-one device, which can be used as an anchor device, but also as a tag device, and can change work mode automatically. It is developed with a UWB chip, a 4G communication module, and a package structure. The ranging accuracy of the UWB chip is about 10 cm, and the positioning accuracy can reach 15~30 cm. When it is regarded as anchor mode, it can measure the distance between the base and itself, or mutual ranging between anchor devices. When it is regarded as tag mode, it can measure the distance between the anchor and itself. The range information will be transmitted to the personnel monitoring and dispatching system with the 4G communication module in real time. The performance as shown in Table 2.

The system is a cloud platform software which has four basic functions. (1) It can receive the positioning data transmitted from the GNSS/UWB base station, and build the positioning datum. (2) It can receive the ranging information transmitted from the UWB anchors, realize the anchor positioning, and then complete the accurate calculation of the location of indoor UWB anchors according to the known points at both ends. (3) It can receive the ranging information transmitted from the UWB tag carried by pedestrian. And the pedestrian trajectory can be calculated. (4) The base station, anchors and pedestrian trajectory are displayed in real time on the system.

### 2.2. Positiong Scheme

**Figure 3:**
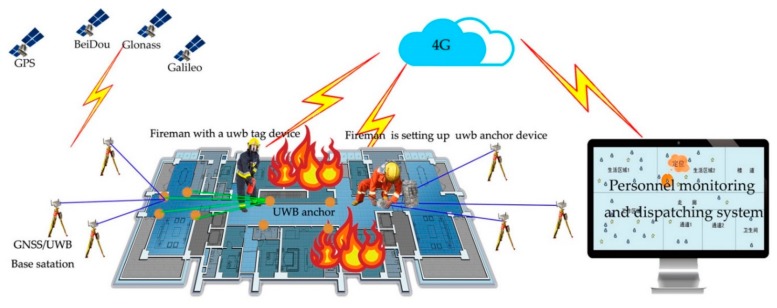
Indoor and outdoor seamless emergency positioning scheme based on GNSS/UWB techniques.

As shown in Figure 3, in an emergency situation, the positioning scheme includes four steps:

Two groups GNSS/UWB base devices are placed at two doors or windows of a building firstly. Every group has there or more devices. Equipment and interior are within line-of-sight range. The device located itself with difference D-GNSS (Difference GNSS) technique. The absolute coordinates of the base are transmitted to the personnel monitoring and dispatching system and regarded as the origin positioning datum.

A firefighter enters the building with a number of tag equipment and place the equipment in corridors and rooms in accordance with certain rules. The first anchor measures the distance between the base station and itself, then transmit to the system, and the approximate coordinates are calculated. Then coordinate of the next anchor will be calculated with base station and the first anchor. In turn, the initial positioning of the anchor is realized. Through the adjustment method of measuring edge network, the accurate position of the anchor is realized. At last, an ad hoc UWB emergency positioning networking will be built.

A fireman wearing a UWB tag device enters the building to put out a fire or rescue. The UWB tag can measure the range between the anchor and itself, and transmit to the system. The move trajectory of the fireman will be calculated.

Through the system, all the positioning trajectories will be showed in real time, which can play a role in the positioning of firefighters and further protects the safety of firefighters.

### 2.3. Technology Roadmap

**Figure 4:**
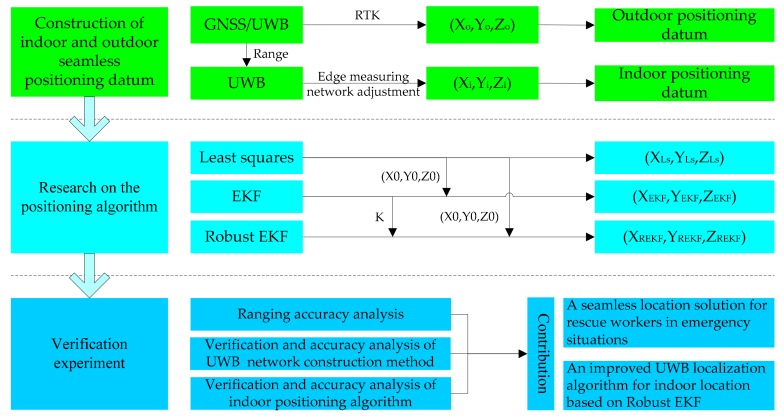
Technology roadmap.

This research aims at the study on an emergency positioning technique, mainly including: the rapid construction method of indoor and outdoor seamless positioning reference under emergency conditions and the indoor positioning algorithm suitable for UWB sensor, and the verified experiments. The technology roadmap is shown in Figure 4, and details are as follows:

First of all, using GNSS/UWB equipment, a spatial reference station with indoor visibility is built outside the building, and the number of reference stations at each entrance or signal transmission port is that three or more make a group for 3-dimension coordinate calculation, two or more make a group for 2-dimension coordinate calculation. The benchmark is located in the mode of GNSS RTK, and the positioning accuracy can reach the centimeter level. In the room, the UWB anchor equipment is placed inwards from one entrance to the other. It is required that more than three devices in the vicinity should see each other, and the placement distance should not exceed the signal transmission distance of the equipment. By using the ranging information collected from each other between the anchor equipment, the coordinates of the anchor are calculated by the edge measuring network adjustment method.

Secondly, a robust EKF algorithm suitable for UWB indoor location is proposed, which can make use of the initial value information provided by least square, and Kalman gain matrix K provided by EKF. The improved algorithm can adjust the size of gain matrix according to the predicted residuals, which can weaken or eliminate the influence of gross errors on the state vector.

Finally, through the simulation of emergency positioning experiment, the UWB ranging accuracy, UWB indoor positioning reference construction method and accuracy, as well as pedestrian movement algorithm and positioning accuracy are verified. The main contributions and innovations are that a seamless location solution for rescue workers in emergency situations and an improved UWB localization algorithm for indoor location based on Robust EKF.

## 3. UWB Positioning Algorithm

### 3.1. UWB Anchor Positioning Algorithm

**Figure 5:**
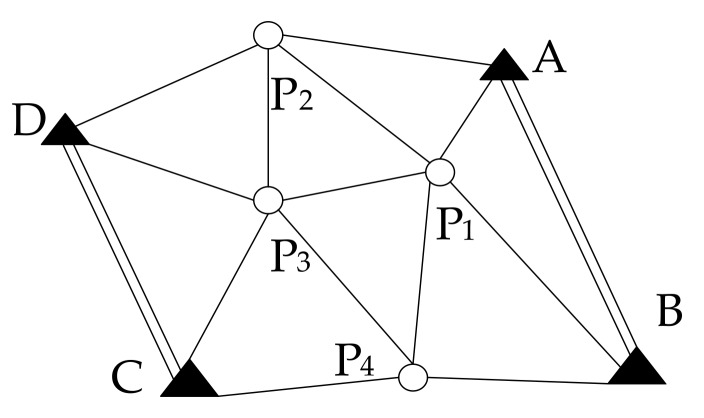
Schematic Diagram of the adjustment of the edge measuring network.

In the process of UWB anchor positioning, taking the distance between the GNSS/UWB station and the UWB anchor as the observation value, and the coordinates of the anchor as unknown parameters, the adjustment of the edge measuring network k is constructed [35]. Figure 5 is a schematic diagram of the adjustment of the edge measuring network. Among them, point A, point B, point C, point D are known points, point P_1_–P_4_ are unknown points. Based on the calculation method of this section, using the coordinates of point A and B and the distance from P_1_ to two points, the coordinates of point P_1_ can be obtained. Then the coordinates of point P_2_ are calculated by using point A and point P_1_, and the coordinates of point P_3_, point P_4_, point C and point D points are calculated by analogy. Because the positions of point C and point D are known, the point P_1_–P_4_ can be adjusted by using the edge measuring network adjustment principle, so that the relatively accurate coordinates of point P_1_–P_4_ can be obtained. If there are more unknown points, their coordinates are also calculated by the above method.

**Figure 6:**
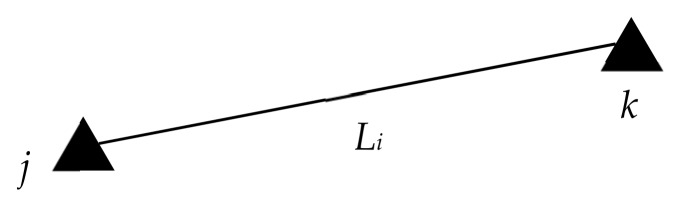
Diagram of side length observation.

Figure 6 is a diagram of the side length observation decomposed by UWB edge-measuring network. *j* and *k* are pending points of anchors. The measured edge length between the anchors is $L_{i}$. Assume that the known edge length between the anchors is ${\hat{L}}_{i}$. The adjusted coordinates of *j* and *k* are ${\hat{X}}_{j}$, ${\hat{Y}}_{j}$, ${\hat{Z}}_{j}$ and ${\hat{X}}_{k}$, ${\hat{Y}}_{k}$, ${\hat{Z}}_{k}$. Assumption:

(1) $$\begin{array}{c} {\hat{X}}_{j}=X_{j}^{0}+{\hat{x}}_{j},{\hat{Y}}_{j}=Y_{j}^{0}+{\hat{y}}_{j},{\hat{Z}}_{j}=Z_{j}^{0}+{\hat{z}}_{j}\\ {\hat{X}}_{k}=X_{k}^{0}+{\hat{x}}_{k},{\hat{Y}}_{k}=Y_{k}^{0}+{\hat{y}}_{k},{\hat{Z}}_{k}=Z_{k}^{0}+{\hat{z}}_{k} \end{array}$$

According to the side length diagram, the adjustment equation ${\hat{L}}_{i}$ can be obtained as follows:

(2) $${\hat{L}}_{i}=L_{i}+v_{i}=\sqrt{{\left({\hat{X}}_{k}-{\hat{X}}_{j}\right)}^{2}+{\left({\hat{Y}}_{k}-{\hat{Y}}_{j}\right)}^{2}+{\left({\hat{Z}}_{k}-{\hat{Z}}_{j}\right)}^{2}}$$

According to the first-order Taylor Equation:

(3) $$L_{i}+v_{i}=S_{jk}^{0}+\frac{\Delta X_{jk}^{0}}{S_{jk}^{0}}\left({\hat{x}}_{k}-{\hat{x}}_{j}\right)+\frac{\Delta Y_{jk}^{0}}{S_{jk}^{0}}\left({\hat{y}}_{k}-{\hat{y}}_{j}\right)+\frac{\Delta Z_{jk}^{0}}{S_{jk}^{0}}\left({\hat{z}}_{k}-{\hat{z}}_{j}\right)$$

where:

(4) $$\begin{array}{c} \Delta {\hat{X}}_{jk}=X_{k}^{0}-X_{j}^{0},\Delta {\hat{Y}}_{jk}=Y_{k}^{0}-Y_{j}^{0},\Delta {\hat{Z}}_{jk}=Z_{k}^{0}-Z_{j}^{0}\\ S_{jk}^{0}=\sqrt{{\left(X_{k}^{0}-X_{k}^{0}\right)}^{2}+{\left(Y_{k}^{0}-Y_{k}^{0}\right)}^{2}+{\left(Z_{k}^{0}-Z_{k}^{0}\right)}^{2}} \end{array}$$

Assumption:

(5) $$l_{i}=L_{i}-S_{jk}^{0}$$

The error equation is

(6) $$v_{i}=-\frac{\Delta X_{jk}^{0}}{S_{jk}^{0}}{\hat{x}}_{j}-\frac{\Delta Y_{jk}^{0}}{S_{jk}^{0}}{\hat{y}}_{j}-\frac{\Delta Z_{jk}^{0}}{S_{jk}^{0}}{\hat{z}}_{j}+\frac{\Delta X_{jk}^{0}}{S_{jk}^{0}}{\hat{x}}_{k}+\frac{\Delta Y_{jk}^{0}}{S_{jk}^{0}}{\hat{y}}_{k}+\frac{\Delta Z_{jk}^{0}}{S_{jk}^{0}}{\hat{z}}_{k}-l_{i}$$

According to Equations (4) and (5), we could calculate the coefficients and constants of the error equation. We can calculate $\Delta X^{0},\Delta Y^{0},\Delta Z^{0},S^{0}$ of all directional side according to the direction of the anchor forward calculation.

(7) $$a=-\frac{\Delta X^{0}}{S^{0}},\;b=-\frac{\Delta Y^{0}}{S^{0}},c=-\frac{\Delta Z^{0}}{S^{0}},l=L-S^{0}$$

According to each side, we could calculate a, b, c coefficient and the constant l. The error model of the whole network can be listed according to Equation (5).

Set

(8) $$B=\left[\begin{array}{c} a_{1}\;b_{1}\ldots \;t_{1}\\ a_{2}\;b_{2}\ldots \;t_{2}\\ \ldots \\ a_{n}\;b_{n}\ldots \;t_{n} \end{array}\right],\;v=\begin{array}{cc} \;[v_{1}\;v_{2}\;\ldots & v_{n} \end{array}{]}^{T}$$

The adjustment equation is

(9) $$V=B\hat{x}-l$$

According to the least square principle, $V^{T}\mathrm{PV}=\min$ and the basic equation is

(10) $$\hat{x}={\left(B^{T}PB\right)}^{-1}B^{T}Pl$$

Then the accurate coordinate value of UWB anchor is

(11) $$\hat{X}=X^{0}+\hat{x}$$

The medium error of unit weight is

(12) $$\sigma_{X}^{2}=\frac{V^{T}PV}{n-t}$$

The RMSE of $\hat{X}$ is

(13) $${\hat{\sigma }}_{x}={\hat{\sigma }}_{0}\sqrt{Q_{x}},{\hat{\sigma }}_{y}={\hat{\sigma }}_{0}\sqrt{Q_{y}},{\hat{\sigma }}_{z}={\hat{\sigma }}_{0}\sqrt{Q_{z}},\hat{\sigma }=\sqrt{{\hat{\sigma }}_{x}{}^{2}+{\hat{\sigma }}_{y}{}^{2}+{\hat{\sigma }}_{z}{}^{2}}$$

### 3.2. UWB Tag Positioning Algorithm

In this paper, the 11-dimensional state vector UWB dynamic navigation model is used as the solution algorithm of UWB positioning results. The state vectors of the EKF model are:

(14) $${\hat{x}}_{k}=\left[\begin{array}{ccccccccccc} \Delta x & \Delta \dot{x} & \Delta \ddot{x} & \Delta y & \Delta \dot{y} & \Delta \ddot{y} & \Delta z & \Delta \dot{z} & \Delta \ddot{z} & \Delta b & \Delta f \end{array}\right]$$

where, Δx,$\;\Delta \dot{x}$,$\;\Delta \ddot{x}$ are the position, velocity and acceleration in X-direction. Y-direction and Z-direction are same to the X-direction. Δb is the clock difference, Δf is the clock drift rate. The acceleration is regarded as a first-order Markov process. The transfer matrix, observation matrix and noise covariance matrix of the corresponding dynamic equation can be derived from Kalman filtering theory and UWB navigation equation.

#### 3.2.1. EKF

The standard Kalman filtering model assumes that the system equation and the observation equation are linear. However, the actual system usually does not meet this assumption. The EKF model can realize the approximate linear realization of the nonlinear system, which can further improve the accuracy of the solution. It is assumed that the nonlinear system is expressed as [36,37]:

(15) $$x_{k}=f_{k-1}\left(x_{k-1}\right)+w_{k}\cdot w_{k}∼N\left(0,Q_{k}\right)$$

(16) $$z_{k}=h_{k}\left(x_{k}\right)+v_{k}\cdot v_{k}∼N\left(0,R_{k}\right)$$

where $x_{k}$ and $x_{k-1}$ are the state vector of k time and k−1 time, respectively. $w_{k}$ and $v_{k}$ are the random noises. $f_{k-1}\left(•\right)$ is a state transition function. $h_{k}\left(•\right)$ is the transfer function between the state vector and the observation vector. $Q_{k}$ is the system dynamic noise variance matrix, and $R_{k}$ is the observed noise variance matrix, that both can be preset [36]. The one-step prediction of discrete extended Kalman filter is as follows:

(17) $${\hat{x}}_{k}\left(-\right)=f_{k-1}\left({\hat{x}}_{k-1}\left(+\right)\right)$$

(18) $${\hat{z}}_{k}=h_{k}\left({\hat{x}}_{k}\left(-\right)\right)$$

The filtering estimation and its corresponding covariance matrix are as follows:

(19) $${\hat{x}}_{k}\left(+\right)={\hat{x}}_{k}\left(-\right)+{\bar{K}}_{k}\left(z_{k}-{\hat{z}}_{k}\right)={\hat{x}}_{k}\left(-\right)+{\bar{K}}_{k}V_{k}$$

(20) $$P_{k}\left(+\right)=\left[I-{\bar{K}}_{k}H_{k}^{\;\left[1\right]}\right]P_{k}\left(-\right)$$

The prediction covariance matrix is:

(21) $$P_{k}\left(-\right)=\Phi_{k-1}^{\;\left[1\right]}P_{k-1}\left(+\right)\Phi_{k-1}^{\;\left[1\right]}{}^{T}+Q_{k-1}$$

The EKF gain matrix is:

(22) $${\bar{K}}_{k}=P_{k}\left(-\right)H_{k}^{\;\left[1\right]T}\;{\left[H_{k}^{\;\left[1\right]}P_{k}\left(-\right)H_{k}^{\;\left[1\right]T}+R_{k}\right]}^{-1}$$

The projected residuals is:

(23) $$V_{k}=z_{k}-{\hat{z}}_{k}$$

The linearized state transition matrix and observation matrix are:

(24) $$\Phi_{k-1}^{\;\left[1\right]}\approx {\left.\frac{\partial f_{k}}{\partial x}\right|}_{x={\hat{x}}_{k-1}\left(-\right)}$$

(25) $$H_{k}^{\;\left[1\right]}\approx {\left.\frac{\partial h_{k}}{\partial x}\right|}_{x={\hat{x}}_{k}\left(-\right)}$$

When the system is nearly linear but not absolutely linear, the EKF can effectively solve the nonlinear problem through a series of approximate calculations, and a better state estimation is given. In addition, because Taylor series expansion only takes the first-order approximation, therefore, the prediction residual does not represent the real observation estimation residual, but it is also enough to describe the dynamic characteristics.

#### 3.2.2. Robust EKF

##### The Influence of Gross Error on the State Estimation of EKF

It is assumed that both the system noise and the observation noise in the EKF model are zero mean white noise. When there is a gross error in the observation, the state estimation will be interfered with. When there are gross errors in the observation vector, the observation equation can be expressed as follows:

(26) $${\tilde{z}}_{k}=h_{k}\left(x_{k}\right)+G_{k}\Delta_{k}+v_{k}$$

$G_{k}$ is a gross error interference matrix, which is composed of elements 0 and 1. If the gross error test passed, the corresponding element of interference matrix $G_{k}$ should be 1, and the gross error is considered as the prediction residual. $\Delta_{k}$ is a gross error vector. The prediction residuals with the influence of gross errors are as follows:

(27) $${\tilde{V}}_{k}={\tilde{z}}_{k}-h_{k}\left({\hat{x}}_{k}\left(-\right)\right)\approx V_{k}+G_{k}\Delta_{k}$$

Here, only the first order term in the dynamic system is considered, and it can be seen that the gross error of the observed value affects the prediction residual. Using Equation (27) instead of Equation (23), the new filtering estimation model is as follows:

(28) $${\tilde{x}}_{k}\left(+\right)={\hat{x}}_{k}\left(-\right)+{\bar{K}}_{k}{\tilde{V}}_{k}$$

Obviously, the gross error in the prediction residual affects the state filtering value through the gain matrix ${\bar{K}}_{k}$. On the basis of robust estimation theory, the influence of gross error on state vector can be weakened or eliminated by adjusting the gain matrix ${\bar{K}}_{k}$ according to the prediction residual. Through the Equation (22), it can be seen that the matrix $H_{k}^{\;\left[1\right]}$ representing the UWB distribution characteristics plays an important role in the determination of the gain matrix ${\bar{K}}_{k}$ [38,39].

##### The Robust EKF Model

Robust EKF process includes equivalent gain matrix construction and iterative solution. Firstly, the equivalent EKF gain matrix is constructed as follows [39]:

(29) $$\left.\begin{array}{cc} {\bar{K}}_{ij} & s_{j}\leq k_{0}\\ {\tilde{K}}_{ij}={\bar{K}}_{ij}\times \frac{k_{0}}{s_{j}}\times {\left[\frac{k_{1}-s_{j}}{k_{1}-k_{0}}\right]}^{2} & k_{0}<s_{j}\leq k_{1}\\ 0 & s_{j}>k_{1} \end{array}\right\}$$

$k_{0}$ and $k_{1}$ are the robust parameters. $k_{0}$ takes 2.5–3.5, $k_{1}$ takes 3.5–4.5.

(30) $$s_{j}=\left|V_{k,j}\right|/\sqrt{r_{j}\sigma_{j}}$$

*i*, *j* are the dimensions of the state vector and the observation vector, respectively. $V_{k,j}$,$r_{j}$ and $\sigma_{j}$ are the prediction residual, redundant observation component and measurement standard deviation of the observation vector j, respectively. The redundant observation components r are determined by the covariance matrix of the geometric distribution of the anchors and the observation vector [40]:

(31) $$r=diag\left(Q_{V_{k}V_{k}}W_{ll}\right)$$

$Q_{VV}$ is the covariance matrix of the residual vector, $W_{ll}$ is the weight matrix of the observation. $diag\left(•\right)$ represents the extraction of diagonal elements of a matrix. Iterative calculations are performed after each update. Given the number of iterations *t*, the state prediction value and the prediction residual are as follows:

(32) $$x_{k,t}\left(-\right)=x_{k,t-1}\left(+\right)$$

(33) $$V_{k,t}=z_{k}-H_{k}x_{k,t}\left(-\right)$$

The state prediction value $x_{k,t}\left(-\right)$ of the *t* time’s iterations is determined by the state filtering value and its prediction residual of the (t − 1) time’s iterations. According to Equations (29)–(31), the equivalent gain matrix is calculated, and the robust filtering value is:

(34) $$x_{k,t}\left(+\right)=x_{k,t-1}\left(-\right)+\tilde{K}V_{k,t}$$

If the difference between $x_{k,t}\left(+\right)$ and $x_{k,t}\left(-\right)$ is less than the given limit difference, the iteration ends. If t = 1, $x_{k,0}\left(+\right)$ is the valuation of the k-time standard EKF. The posterior covariance matrix is:

(35) $$P_{k}\left(+\right)=\;\left[I-{\tilde{K}}_{k,t}H_{k}^{\;\left[1\right]}\right]P_{k}\left(-\right)$$

${\tilde{K}}_{k,t}$ is the final equivalent Kalman filter gain matrix at the end of the iteration.

##### Improved Robust EKF

If the robust Kalman filter model is used as the standard model of UWB navigation, it is necessary to carry out robust iteration for each epoch, so as to reduce the speed of navigation solution. In this paper, the statistical method is used to determine whether there is a gross error, if so, the robust EKF model is called; if it does not exist, EKF is directly used to navigate and solve the problem. The prediction residual in Equation (23) is the m-dimensional zero mean, that is:

(36) $$E\;\left[V_{k}]=0\;[V_{k}V_{k}^{T}\right]$$

The updated covariance $Q_{V_{k}V_{k}}$ is:

(37) $$Q_{V_{k}V_{k}}=H_{k}^{\;\left[1\right]}P_{k}\left(-\right)H_{k}^{\;\left[1\right]T}+R_{k}$$

The statistical test was

(38) $$\lambda_{k}\left(m\right)=\left(V_{k}^{T}\right)\left(Q^{-1}{}_{V_{k}V_{k}}\right)\left(V_{k}\right)$$

When there is no gross error, the statistical test $\lambda_{k}\left(m\right)$ obeys the $\chi^{2}$ distribution with degree of freedom *m*. if there is a gross error, the statistical test $\lambda_{k}\left(m\right)$ obeys the non-central $\chi^{2}$ distribution with degree of freedom m. m denotes the dimension of the observation vector. The critical value $T_{D}$ of gross error detection is determined by the test $\chi^{2}$ of significance level α, and the criteria are as follows:

(39) $$\left\{\begin{array}{c} \lambda_{k}>T_{D},\mathrm{when}\;\mathrm{there}\;\mathrm{is}\;\mathrm{an}\;\mathrm{anomaly}\\ \lambda_{k}\leq T_{D},\mathrm{when}\;\mathrm{there}\;\mathrm{is}\;\mathrm{no}\;\mathrm{anomaly} \end{array}\right.$$

When the statistical test shows that there are gross errors, the robust EKF model is called for navigation solution, which achieves the purpose of improving the efficiency of the model operation.

## 4. Experimental Verification

### 4.1. Introduction of Experimental Scene

**Figure 7:**
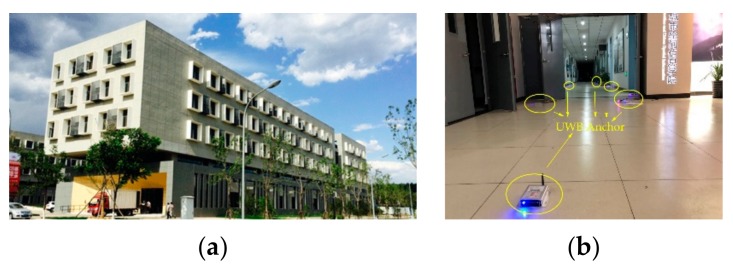
Pedestrian indoor positioning experiment scene. (**a**) Outside scene of the building (**b**) The scene of the corridor.

In order to verify the effectiveness of the proposed scheme, a field experiment was carried out in the first floor of School of Geomatics and Urban Spatial Informatics of Beijing University of Civil and Architectural, Beijing, China. The length of the corridor is about 65 m, and the width is about 3 m. The area of the laboratory is about 6 m × 8 m. Because there is an equipment room at the end of the corridor that is closed, so the foyer of the building, half of the corridor and two laboratories are used in the laboratory, shown in Figure 7.

**Figure 8:**
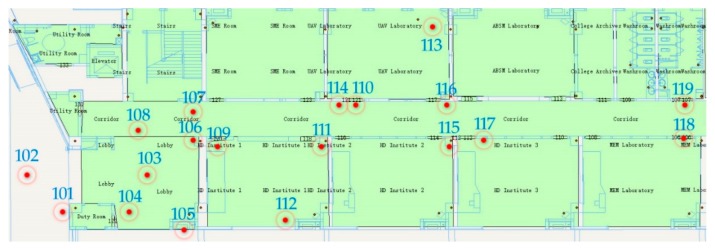
Indoor Map and the approximate position UWB anchors on the first floor of the building.

Because only the plane coordinates are calculated in the course of the experiment, so just two GNSS/UWB devices are used in each entrance and exit, as shown in Figure 8. First of all, a group of GNSS/UWB base stations are placed at the entrances and exits of the building. The coordinates of the base station of point 101 and point 102 are obtained by D-GNSS technique used as the outdoor positioning reference. Because the equipment room is closed, the other two reference points cannot be obtained by GNSS/UWB equipment. So, the coordinates of another two points are collected by total station, as shown in point 118 and point 119. Then, 15 UWB anchor equipment are placed in the corridor on the first floor and in two laboratories. During the placement process, the anchor should be kept within the line-of-sight with at least two surrounding anchors.

UWB equipment has two working modes: anchor and tag. In the tag mode, the device will request ranging information for 10 times to three or more anchor within the line of sight, and then automatically switches to the anchor mode. The distance is used as the observation value of the anchor coordinate solution. The coordinates of the anchors are calculated by the adjustment of the edge measuring network. After the coordinates of the anchors are obtained, an experimenter carries the tag equipment and walk along the fixed route. The distance between the anchors is collected by the tag and transmitted to the cloud platform in real time to solve the pedestrian location.

In order to analyze the accuracy of ranging and positioning, the precise coordinates of each anchor and the real trajectory of the motion are measured in advance.

### 4.2. The Positioning Results of the UWB Anchors

Due to the influence of indoor signal occlusion, multi-path and other factors, UWB ranging information will have errors. Ranging error has a great influence on the adjustment results of edge measuring network, especially in the narrow and long measuring network. In order to obtain better UWB ranging accuracy, the average of multiple ranging information is taken as UWB ranging value.

**Figure 9:**
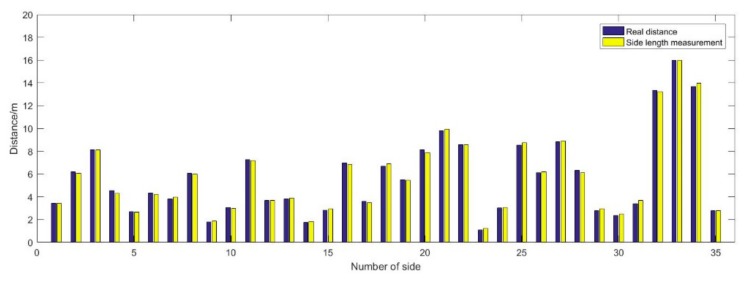
Comparison between mean distance measured by UWB and Real distance.

Figure 9 shows the comparison between the real distance and the measurement distance. There are 19 anchors in total, and 85 sides are acquired. Among them, 35 adjacent edges are used to construct edge-measuring meshes, shown in Figure 11. 

**Figure 10:**
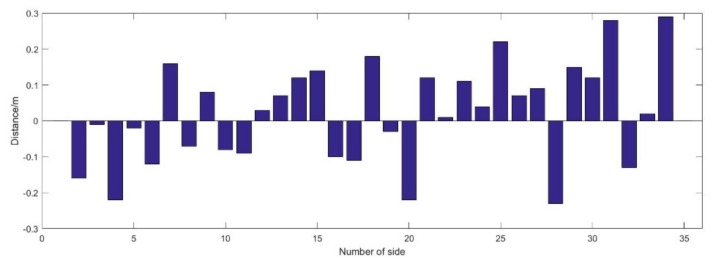
UWB ranging error.

From Figure 10, it can be seen, the maximum ranging error is 0.29 m, and the minimum ranging error is 0.01 m, except for the first and the last side of which the distance is real distance calculated with given points. The mean ranging error is 0.02 m, the variance of the ranging error is also 0.02 m. Although, the mean error and the variance is very small, there are 6 sides that the absolute ranging error is more than 0.2 m, and the RMSE of ranging error is the order of 0.15 m.

**Figure 11:**
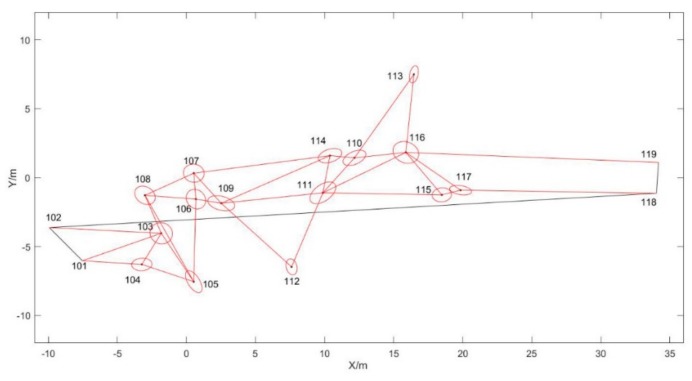
The side network adjustment results of UWB anchors.

Figure 11 shows the results of the side network adjustment of UWB anchors. Among them, point 101, point 102, point 118 and point 119 are given points. The coordinates of point 101 and point 102 are acquired with GNSS/UWB base station device, and the coordinates of point 118 and point 119 are acquired by total station. In order to display conveniently, the coordinate system is changed to local coordinate system. Point 103–117 are calculated with the method of side network adjustment. The circle around the point is the error ellipse of the point.

**Figure 12:**
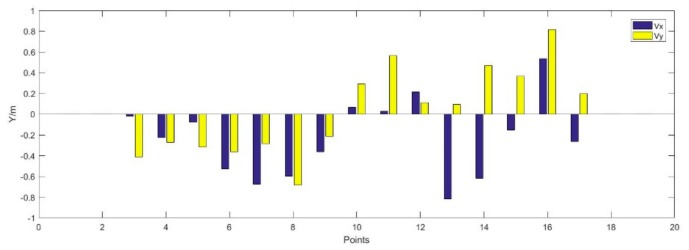
Error of the UWB anchors after adjustment.

Figure 12 shows the error of the coordinates of the UWB anchors after adjustment. The maximum and minimum errors in X direction are −0.82 m and −0.01 m, and the maximum and minimum errors in Y direction are 0.82 m and 0.09 m. The RMSE of the errors are 0.31 m and 0.36 m in X and Y directions.

### 4.3. Pedestrian Motion Positioning Results

**Figure 13:**
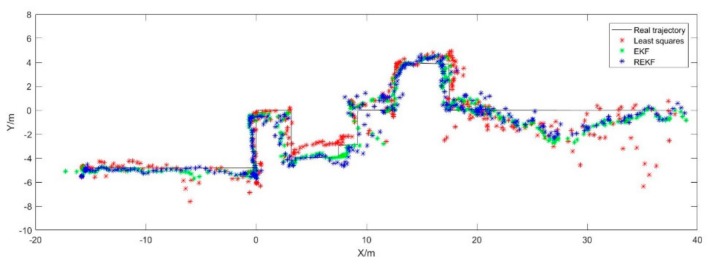
The motion trajectories of the pedestrian calculated with three algorithm.

It can be seen from Figure 13, generally speaking, the positioning algorithm of REKF (Robust EKF) has the best accuracy, followed by EKF and least squares. From a local point of view, the range from −15 m to 0 m and 12 m to 22 m, the positioning accuracy is high, that is because of that the number of anchors with good visual condition is large and the location distribution is reasonable. The range from 0 m to 12 m and 22 m to 35 m, the accuracy is lower than others, which is because of that the number of anchors is small, and the accuracy of point 11 and point 16 is worse.

**Figure 14:**
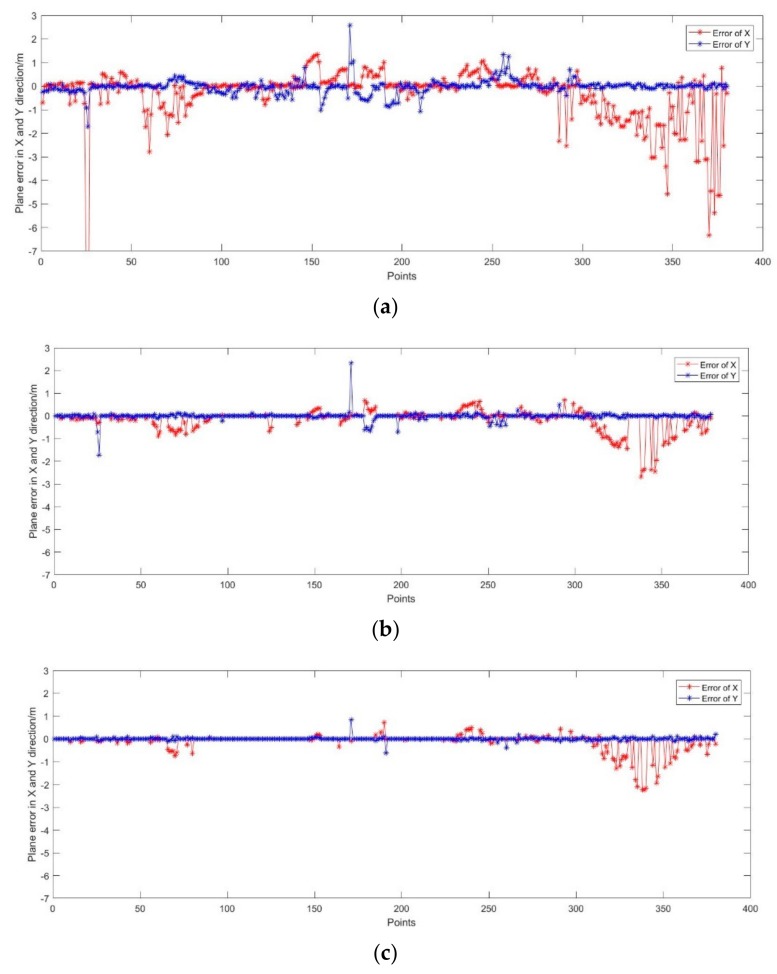
Plane error calculated based on three filtering algorithm. (**a**) Plane error calculated based on least square algorithm; (**b**) Plane error calculated based on Extended Kalman Filter (EKF) algorithm; (**c**) Plane error calculated based on Robust-EKF algorithm.

Because the real trajectory is a curve fitted by measured discrete locating points, in order to analyze the accuracy of UWB location, the curve is linearly interpolated into checkpoints consistent with UWB points. The location accuracy of UWB point is determined by comparing the UWB point with the nearest checkpoint. Figure 14 shows the positioning error of three algorithm. From Figure 14a, the maximum and minimum positioning error are −15.64 m and 0 m in X direction, the maximum and minimum positioning error are 2.58 m and 0 m in Y direction. The RMSE is ±1.35 m and ±0.31 m in X and Y direction respectively. From Figure 14b, the maximum and minimum positioning error are −2.69 m and 0 m in X direction, the maximum and minimum positioning error are 2.32 m and 0 m in Y direction. The RMSE is ±0.44 m and ±0.18 m in X and Y direction respectively. From Figure 14c, the maximum and minimum positioning error are −2.24 m and 0 m in X direction, the maximum and minimum positioning error are 0.85 m and 0 m in Y direction. The RMSE is ±0.36 m and ±0.07 m in X and Y direction respectively.

In addition, although the least squares method can be used to solve the location problem, the discrete degree of the location error is the greatest, and the effect of restraining the maximum of the location error is not satisfactory. The EKF method has been improved to some extent, especially in the X direction. The REKF method achieves better improvement in both directions. In total, the plane positioning RMSE based on least square algorithm is the order of 1.43 m. The plane positioning RMSE based on EKF algorithm is the order of 0.48 m. The plane positioning RMSE based on Robust-EKF algorithm is the order of 0.38 m.

**Figure 15:**
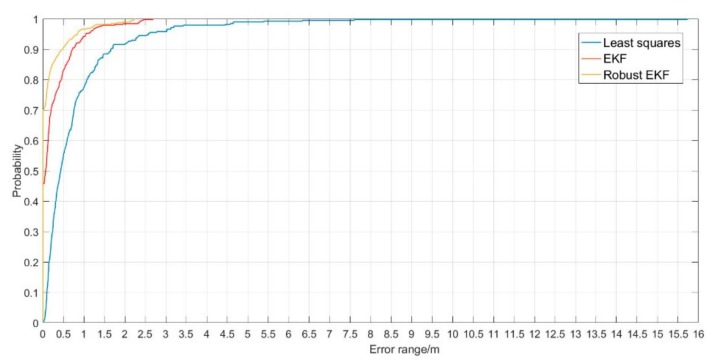
The cumulative distribution of plane error calculated based on three filtering algorithm.

Figure 15 is a map of the probability distribution of plane errors calculated by three positioning methods. It can be seen that based on the robust EKF algorithm, 70% of the point location error is 0 m, 90% of the point location error is better than 0.50 m, and 10% of the point location error is in the range of 0.50 m and 2.50 m. Based on EKF algorithm, about 46% of the point location error is 0 m, 80% of the point location error is in the range of 0.50 m, and 20% of the point location error is in the range of 0.50 m and 2.80 m. Based on the least square algorithm, 50% of the point location error is in the range of 0 to 0.5 m, 30% of the point location error is in the range of 0.5 m to 1.0 m, 20% of the point location error is more than 1 m, and the maximum location error is about 15.6 m. In summary, no matter the positioning accuracy or error distribution, the positioning result of robust EKF algorithm is the best, EKF is the second, and the least square accuracy is the lowest.

The summaries of this research are as follows:

(1) This paper innovatively proposes a seamless location solution for rescue workers in emergency situations. Outdoor location can be achieved by D-GNSS technology, and indoor location can be realized by ad hoc UWB location network. The biggest advantage of this scheme is that it does not need to set up UWB anchors and measure their positions in advance, and the anchor coordinates are iterated to the interior through the outdoor positioning data. So this scheme is suitable for rapid deployment in emergency situations.
(2) When the UWB anchors are placed in anchor mode, the location of three or more nearby anchors are kept in the condition line-of-sight, and the location of the anchor is reasonably distributed as far as possible. The adjustment algorithm of edge network requires high accuracy of edge length, so enough redundant observations are maintained to ensure that the ranging accuracy is better than 0.2 m as far as possible. The proposed progressive UWB base station placement method can realize the UWB emergency network nodes location of the order of 0.35 m.
(3) Another contribution of this paper is that an improved UWB localization algorithm for indoor location based on Robust EKF is proposed. The improved algorithm can adjust the size of gain matrix according to the predicted residuals, which can weaken or eliminate the influence of gross errors on the state vector. Comparing with EKF and least squares method, the Robust EKF method has higher positioning accuracy that the plane accuracy up to the order of 0.38 m. Compared to the standard EKF and least square algorithm, the proposed algorithm improves the positioning accuracy by 20.83% and 73.43% compared with, respectively.

## 5. Conclusions

In order to solve the problem of fast and accurate positioning of rescue workers under emergency conditions, a seamless indoor and outdoor positioning solution based on ad hoc GNSS/UWB network is proposed in this paper. First of all, we can use GNSS/UWB equipment to quickly build high-precision spatial benchmark around the building based on D-GNSS technology. Secondly, the UWB indoor positioning network is constructed using the recurrence method, and the initial position of UWB base station is calculated in real time through the cloud platform, and then the edge measuring network adjustment method is used to adjust the positioning accuracy of UWB anchors. Finally, based on the robust EKF positioning algorithm, it provides high-precision location information for indoor rescue workers. The experimental results show that the solution is robust and reliable, and that the indoor positioning accuracy can be decimeter-level. This scheme not only can provide the high accuracy position datum, but also can solve the positioning problem of a rescuer in real time. So, the scheme is adaptive to indoor emergency rescue. However, because of the problem of the signal occlusion and multipath effects, too many UWB anchors are needed and attention should be paid to the reasonable layout of the anchor network, which will decrease the efficiency. Therefore, further research will focus on solving the problem of UWB network shape optimization design and introducing auxiliary sensors such as INS to reduce the number of UWB anchors.

## Figures and Tables

**Table 1:** Performance of GNSS/UWB seamless positioning base device.

Performance	Parameter
The receiving sensitivity of UWB	−118 dBm
The ranging accuracy of UWB	≤10 cm
The ranging distance in line-of-sight	Max 880 m
Satellite system	GPS, BDS, Glonass, Galileo
Frequency	B1/B2/B3/L1/L2/L5/G1/G2
RTK positioning accuracy	2–5 cm
Single point positioning accuracy	≤1.5 m
The sampling rate of GNSS	1–10 Hz
Communication mode	4G

**Table 2:** Performance of UWB anchor and tag two-in-one equipment device.

Performance	Parameter
Size	9 × 12.5 × 0.7 cm
Operating voltage	3–5 V
Receiving sensitivity	−118 dBm
Ranging accuracy	≤10 cm
Line-of-sight ranging distance	Max 880 m
Positioning accuracy	≤30 cm
Positioning sampling rate	1–5 Hz
Communication mode	4G
Standard hardware interface	USB, UART, I2C, SPI, GPIO
